# Decentralizing the future: Value creation in Web 3.0 and the Metaverse

**DOI:** 10.12688/openreseurope.20906.1

**Published:** 2025-08-06

**Authors:** Guido Perboli, Francesca Merlo, Chiara Vandoni

**Affiliations:** 1 DIGEP, Politecnico di Torino, Turin, Piedmont, 10135, Italy

**Keywords:** Web 3.0, Metaverse, Blockchain Governance, Data Sovereignty

## Abstract

The emergence of Web 3.0 and the Metaverse marks a transformative shift in the evolution of the internet and digital ecosystems. This paper explores the foundational principles of decentralization, user autonomy, and data transparency that underpin Web 3.0 technologies, including blockchain, smart contracts, and digital wallets. We analyze how these innovations are reshaping business models, enabling new forms of value creation, and redefining digital ownership and governance. In parallel, we examine the Metaverse as a virtual, immersive environment integrating Web 3.0 infrastructure, and its potential to revolutionize sectors such as logistics, education, finance, and data management. The study also highlights the critical role of a holistic framework encompassing technological, economic, and legal pillars. A special focus is given to data provenance, privacy-preserving computation, and the need for coherent regulatory strategies in light of GDPR, the AI Act, and the Data Act ( European Parliament, 2016; European Parliament, 2023; European Parliament, 2024). Finally, we identify emerging challenges related to NFT authenticity, system sustainability, and user experience, proposing a multidisciplinary and lean governance approach to guide future developments.

## Plain language summary

The internet is entering a new phase called Web 3.0, which emphasizes decentralization, transparency, and giving people more control over their data. Unlike today's internet, dominated by big companies, Web 3.0 uses technologies like blockchain and smart contracts to spread control among users, making the internet fairer and more secure. This shift allows users to own their data, protect their privacy, and benefit economically from the content they create. Alongside Web 3.0, the Metaverse is emerging as a virtual world where people can interact, work, and play through immersive experiences. It uses blockchain to ensure digital items, like artwork or virtual land, can be owned and traded securely. This opens up new business opportunities and can also help the environment by creating digital versions of physical goods, reducing waste. However, these technologies face challenges, including energy consumption, data security, and the need for clear regulations. Ensuring sustainability, economically, socially, and environmentally, is crucial. A unified approach that includes legal frameworks, economic strategies, and technological standards will be necessary to create an inclusive, safe, and trustworthy digital future.

## 1 Introduction

The rapid evolution of the digital landscape has brought Web 3.0 and the Metaverse to the forefront of both technological innovation and strategic business discourse. While much of the mainstream attention has focused on cryptocurrencies, NFTs, and immersive virtual experiences, a growing body of research and experimentation is beginning to position these technologies within a broader framework of sustainability. In this context, sustainability extends beyond environmental concerns to encompass economic resilience, social inclusion, and governance transparency, key pillars of long-term digital equity and value creation.

Web 3.0, often described as the next generation of the internet, introduces a paradigm shift grounded in decentralization, interoperability, and user empowerment, promising a more equitable digital infrastructure compared to Web2’s centralized platforms that control data and extract value from user interactions by distributing data ownership and governance across peer-to-peer networks. These characteristics open up new possibilities for creating sustainable digital ecosystems, where value is co-created and shared transparently, and where resources, digital, computational, and even human, are allocated more efficiently.

From a sustainability standpoint, the architecture of Web 3.0 offers several structural advantages. The use of distributed ledger technologies (DLTs) enables greater traceability and accountability in value chains, which is particularly relevant in sectors like logistics, manufacturing, and energy. Smart contracts can automate and enforce environmental and social compliance in supply chains, while digital identities and verifiable credentials can support ethical sourcing and responsible consumption. Furthermore, the shift toward self-sovereign identity and data sovereignty empowers individuals to manage and monetize their own data, reducing dependency on monopolistic intermediaries and mitigating privacy and security risks.

In parallel with Web 3.0, the Metaverse is emerging as a virtual environment where users can interact, work, trade, and create through immersive digital experiences. When integrated with blockchain and Web 3.0 infrastructure, the Metaverse has the potential to enable circular digital economies, where digital assets are reusable, interoperable, and verifiably owned. For instance, the creation of digital twins of physical goods or environments can reduce material waste, support predictive maintenance, and enable efficient simulation of real-world processes. These capabilities can lead to dematerialization, the replacement of physical resources with digital equivalents, thus contributing to reduced carbon emissions and more sustainable consumption patterns.

However, the sustainability of the Metaverse also raises critical challenges. The infrastructure required to support immersive experiences and decentralized computing can be energy intensive. Therefore, the deployment of green computing practices, energy-efficient consensus mechanisms (e.g., Proof of Stake over Proof of Work), and integration with renewable energy sources becomes crucial. Sustainable Metaverse development must be guided by life-cycle assessments, considering not only the environmental footprint of infrastructure but also the social implications of digital inclusion, accessibility, and data ethics.

The main objective of this paper is to provide a clear explanation of the concepts of Web 3.0 and the Metaverse, examining the business models and value creation strategies associated with this new digital era. Furthermore, we emphasize the importance of developing a holistic approach that encompasses legal, economic, and technological frameworks, with particular focus on the crucial role of the legal framework, which is often overlooked in existing literature.

Despite growing interest in Web 3.0 and the Metaverse, the challenges that lie ahead are significant: a universally accepted definition is still lacking, and the unresolved complexities surrounding various constructions of Web 3.0 make the development of a legal framework increasingly necessary.

Recognizing the transformative potential of Web 3.0 and the Metaverse requires an integrated approach that balances innovation with regulation, autonomy with responsibility, and growth with resilience. This chapter proposes a holistic sustainability framework that integrates three core dimensions:

1. Technological Sustainability – ensuring that digital infrastructure (blockchain, smart contracts, decentralized storage) is scalable, secure, and energy-efficient.
2. Economic Sustainability – fostering business models that reward participation, enhance transparency, and reduce friction through token economies and decentralized finance (DeFi).
3. Legal and Governance Sustainability – establishing clear rules and accountability for data ownership, digital identity, and rights enforcement, especially in light of evolving regulatory contexts such as the GDPR, the Data Act, and the AI Act.

The transition to a sustainable Web 3.0 and Metaverse is neither automatic nor free from friction. Many organizations are currently engaging with these technologies through pilot projects or limited experiments, often without a clear long-term strategy or understanding of the systemic implications. Without proper legal infrastructure, coherent economic models, and user-friendly technological solutions, the risk is that these innovations remain speculative or exclusionary. Moreover, the absence of standardization frameworks for use case definition, data interoperability, and value measurement hinders broad adoption and scalability.

Ultimately, the promise of Web 3.0 and the Metaverse lies in their potential to realign digital transformation with sustainable development goals. By reducing dependencies on centralized platforms, enabling greater individual agency, and supporting transparent, automated, and auditable systems, these technologies can contribute to a more inclusive and sustainable digital future. This paper aims to explore that potential, identify critical gaps, and offer guidance for stakeholders, both public and private, interested in shaping a Web 3.0 ecosystem that is not only innovative but also resilient, ethical, and sustainable ( Lage *et al.*, 2022).

The remainder of the paper is structured as follows. Section 2 introduces the key concepts and economic dynamics of Web 3.0 and the Metaverse, highlighting their evolution, technological underpinnings, and potential for value creation. Section 3 provides a technical analysis of the foundational components of Web 3.0, including blockchain, digital wallets, and smart contracts. Section 4 explores real-world use cases, identifying emerging business models and examining how organizations are leveraging Web 3.0 technologies to create value, with a particular focus on the interplay between technological, economic, and legal pillars. Section 5 discusses managerial insights and future developments, emphasizing the importance of a unified, sustainability-oriented framework and addressing critical issues such as data marketplaces, NFTs, and regulatory integration. Finally, Section 6 offers concluding remarks, summarizing the key findings and proposing a multidisciplinary path forward for a resilient and inclusive Web 3.0 and Metaverse ecosystem.

## 2 Web 3.0 and the metaverse

Web 3.0, also known as the “third generation of the Internet,” marks a fundamental turning point in the history of the World Wide Web ( Huang *et al.*, 2023). This new era was ushered in by the introduction of distributed ledger technologies and blockchain. In contrast to the dominance of large corporations such as Google, Facebook, and Amazon in Web 2.0, Web 3.0 is built on core principles of decentralization, user control, and transparency.

At the heart of Web 3.0 lies the concept of decentralization, individuals or single entities no longer own services, but instead belong to the entire network. This eliminates intermediaries, making the web more resistant to censorship and external control. Another distinctive feature is user control: users now have full ownership of their data and assets through self-custodial digital wallets. This translates into increased privacy and security, allowing individuals to manage their information autonomously.

Blockchain technology, with its guarantees of traceability and immutability of data and transactions, brings greater transparency and security to Web 3.0. As a result, the web becomes a more trustworthy and secure space for all users. This new paradigm offers several potential benefits, including better use of economic resources across various sectors, greater access and control for users, and significant innovations in business models and applications. Assuming that assets are collectively held by the network, three main commercial approaches can be identified ( Black, 2021):

- Virtual products: Many brands are developing new products for Web 3.0, often in collaboration with established virtual goods producers and digital platforms. In this model, companies retain full control over the assets, including their appearance and functionality in virtual environments and digital wallets. These experiments attract younger audiences and provide valuable behavioral insights.
- Hybrid products: This approach enhances physical products with digital information recorded on the blockchain, granting each item a unique identity. It allows brands to prevent counterfeiting and benefit from secondary market sales, something traditionally difficult to achieve.
- Distributed ownership: In this brand governance model, instead of selling an item to a single customer, a company allows multiple users to share ownership via blockchain. This strategy helps brands gain real-time insights into consumer demand, improving negotiations with producers and securing better prices for consumers.

Web 3.0 is expected to have a significant impact across various sectors ( EY & Luiss, 2023), including manufacturing and logistics, data management and valorization, and the implementation of business development policies and innovation strategies. It may foster a new environment for online education, using personalized technologies to enhance learning effectiveness. In the business world, Web 3.0 could revolutionize how companies use collected data (such as market and customer data) and enable the creation of new products and business models with lower transaction costs and greater trust through decentralized consensus mechanisms. Decentralization fosters greater economic efficiency, while increased democratization of the web leads to a fairer distribution of power and access to online resources. Moreover, the rise of new sectors like decentralized finance (DeFi), decentralized social media, and the Metaverse paves the way for new business opportunities and modes of online interaction.

However, despite its promises, Web 3.0 still faces several challenges. The underlying technology is still evolving and can be complex for non-expert users. Additionally, the absence of a clear regulatory and legal framework at a global level creates uncertainty for developers and companies operating in this space.

The Metaverse is emerging as a virtual environment that merges the Internet, Virtual Reality (VR), Augmented Reality (AR), and digital worlds, allowing users to interact with digital objects through avatars. This imagined space aims to seamlessly blend digital and physical realms for social interaction, entertainment, work, and commerce. As it evolves, the Metaverse is expected to integrate technologies such as blockchain, artificial intelligence, and advanced network infrastructures to offer users a more sophisticated, immersive, and interactive experience.

The term “Metaverse” first appeared in the 1992 science fiction novel *Snow Crash* and has since evolved from narrative fiction to a transformative force in the virtual reality landscape ( Huang *et al.*, 2023). In recent years, growing interest from researchers and developers has turned the Metaverse into a dynamic field of exploration. It has now entered a phase of rapid development. 2021 is often regarded as the first true year of the Metaverse, as it gained widespread recognition and debate. Public interest was sparked by Roblox’s IPO on March 11, 2021, bringing the Metaverse to mainstream attention. Tech giants like Facebook, which rebranded as Meta to reflect its Metaverse focus, began outlining their plans. This iconic event triggered a Metaverse boom among major technology players. Two key motivations identified by W.Y.B. Lim *et al.* (2022) fuel this enthusiasm: post-COVID-19 social changes and technological advancements that support its growth. Tech companies such as Facebook, Microsoft, Tencent, and ByteDance have continued to invest heavily in the Metaverse, expanding their presence in related sectors such as VR/AR hardware, 3D game engines, and content creation platforms. Additionally, the rise of NFTs has directly contributed to the Metaverse boom ( Guidi & Michienzi, 2023).

At the core of the Metaverse lies a shared vision: a massively scaled, interoperable network of real-time rendered 3D virtual worlds ( Sadowski & Beegle, 2023). This vision includes a spectrum of data, identity, history, rights, assets, communication, and payments. The economic ecosystem surrounding the Metaverse is expanding rapidly, with a stark contrast in market capitalization between Web 2.0 and Web 3.0 companies. This transformation is underpinned by the Web 3.0 approach, which gives users full control of their digital assets through blockchain and tokenization. As envisioned by tech entrepreneurs, the Metaverse appears as a computer-generated virtual world, a frontier in social computing, destined to impact multiple facets of society.

As Web 3.0 and the Metaverse continue to evolve, their economic foundations emerge as a driving force of innovation. Closely linked to a blockchain-based economic model, the Metaverse holds significant financial potential for investors and users ( Cheng, 2023). Blockchain, as the decentralized infrastructure of Web 3.0, enables transparent and traceable digital asset trading. Smart contracts allow programmability and automated execution of transactions, while NFTs empower stakeholders with ownership rights and participation in the Metaverse’s economic value. The question of how economic systems should be structured in the Metaverse is central, as these systems serve as the foundation for nearly all activity within it, shaping its economic dynamics:

- Digital creation: The Metaverse economy relies on user creativity and has become a promising research field, pushing innovation in this virtual dimension. Users play a crucial role as providers of data and resources, shaping a world where they own a wide range of digital assets. Incentive mechanisms are essential to support user contributions and motivate all stakeholders to engage with the Metaverse economy. These incentives sustain the economic cycle of digital assets. Metaverse users create significant volumes of content, including user-generated content (UGC), AI-generated content (AIGC), and professionally generated content (PGC), either independently or with AI assistance. Users actively participate in economic activities like creation, exchange, and investment to generate income. Without proper incentives, users may hesitate to contribute resources or data due to privacy concerns.
- Digital assets: These fall into two main categories: digital currencies and digital tokens. Digital currencies include government-issued currencies like the digital euro, and cryptocurrencies like Bitcoin and Ethereum issued by decentralized blockchain systems. Digital tokens, on the other hand, are digital representations of assets and can be fungible or non-fungible (NFTs). Fungible tokens, such as utility tokens, are interchangeable with others of the same type and value, whereas NFTs are unique and tied to specific assets, such as digital artworks or collectibles ( Joy *et al.*, 2022).

These digital tokens can serve various functions, representing physical, abstract, or virtual goods. Their fungibility is key: fungible assets can be swapped for identical ones, while NFTs are unique and non-replicable, with value tied to the specific asset they represent. All digital assets benefit from blockchain technology, which guarantees immutability, traceability, and security. Cryptocurrencies and NFTs are decentralized, and their value is determined by the market, while central bank digital currencies are government-regulated and tend to be more stable in value. The combination of these digital asset types helps define the Metaverse’s economic landscape, offering users opportunities to participate and invest in a highly innovative and dynamic context.

## 3 Technology analysis

To fully understand the concept of the Metaverse, it is essential to examine the core technologies that underpin Web 3.0, namely blockchain, digital wallets, and smart contracts (see Capocasale & Perboli, 2022; Capocasale *et al.*, 2023; Capocasale, *et al.*, 2025 for a more detailed discussion).

### 3.1 Blockchain infrastructure

Blockchain infrastructure represents the cornerstone of Web 3.0 architecture, offering a decentralized and tamper-resistant mechanism for recording and verifying transactions across a distributed network. Unlike traditional centralized databases, which rely on a trusted authority for validation and access control, blockchain networks achieve consensus through cryptographic protocols and algorithmic agreement among participants. This decentralization reduces reliance on intermediaries and enhances transparency, as all transactional data is visible, immutable, and auditable across the ledger.

The choice of consensus mechanism greatly affects a blockchain's properties. Proof of Work (PoW), used for example by Bitcoin, ensures strong security but consumes a lot of energy, posing environmental concerns. Proof of Stake (PoS), adopted by Ethereum post-"Merge," offers substantial energy efficiency while preserving security. Variants like Delegated Proof of Stake (DPoS) and Proof of Authority (PoA) are designed for specific needs, such as private or consortium blockchains, focusing on scalability and faster transactions.

Blockchain infrastructure also facilitates the tokenization of digital and physical assets, enables programmable financial instruments, and supports decentralized governance mechanisms. These functions are central to the operational models of both Web 3.0 platforms and Metaverse environments, where blockchain ensures the verifiability of digital ownership and the integrity of interactions within and across virtual spaces. Moreover, blockchain’s capacity to function as a trustless infrastructure—where parties can transact without prior trust or intermediaries—constitutes one of the most transformative aspects of this technology, especially in the context of global, interoperable digital ecosystems.

### 3.2 Digital wallets and identity management

Digital wallets are critical to the user experience in Web 3.0 ecosystems, functioning as gateways for accessing, managing, and transferring digital assets. These software or hardware-based tools store users' cryptographic keys, which are essential for signing transactions, proving ownership, and interacting securely with decentralized applications. Wallets can be classified into self-custodial and custodial types, each presenting distinct trade-offs in terms of control, security, and user responsibility.

Self-custodial wallets grant users full control over their private keys, thereby aligning with the core principle of user sovereignty that underpins Web 3.0. However, they also place the burden of security and key management entirely on the user, which may hinder adoption among non-experts. Custodial wallets, by contrast, delegate key management to a trusted third party, offering greater convenience but reintroducing a level of centralization that can be antithetical to Web 3.0’s decentralization ethos.

Beyond asset management, wallets are increasingly becoming instruments of identity in the digital realm. Emerging frameworks such as Decentralized Identifiers (DIDs) and Verifiable Credentials (VCs), supported by the W3C, are redefining identity management by enabling users to authenticate themselves without relying on centralized authorities. These technologies allow for selective disclosure of personal data, enhancing privacy and reducing the risks associated with data breaches and identity theft. Within the context of the Metaverse, wallets not only manage tokens and credentials but also serve as persistent identity anchors that span multiple virtual environments, thereby facilitating cross-platform interaction and reputation building.

### 3.3 Smart contracts

Smart contracts are self-executing code deployed on a blockchain, programmed to carry out specific actions when predefined conditions are met. These digital contracts eliminate the need for intermediaries in transactional processes, enabling secure, automated, and transparent interactions across decentralized systems. By embedding logic directly into the blockchain, smart contracts ensure that agreements are executed exactly as intended, without reliance on human discretion or third-party enforcement.

The deployment of smart contracts has enabled the rise of decentralized applications (dApps), which provide services ranging from decentralized finance (DeFi) to digital art marketplaces and decentralized autonomous organizations (DAOs). Their benefits include increased operational efficiency, reduced transaction costs, and improved auditability. In particular, the immutability of smart contracts presents both strengths and limitations while enhancing trust and reliability. Once deployed, the contract’s code cannot be altered, which secures it against tampering but also complicates the process of correcting bugs or updating contract logic. This has led to the adoption of upgradable smart contract architectures, such as proxy patterns, which allow limited modification through predefined governance mechanisms.

Smart contracts are also central to the operation of the Metaverse. They manage ownership and transfer of digital assets (including NFTs), automate complex in-game economies, and enforce access control and licensing schemes. However, their effectiveness depends on accurate input data, secure coding practices, and careful legal interpretation. The legal status of smart contracts remains an evolving issue, with some jurisdictions beginning to recognize their binding nature under certain conditions, while others still grapple with the implications of code-as-law.

### 3.4 Decentralized storage

The need for reliable and scalable data storage is particularly acute in Web 3.0 and Metaverse ecosystems, where digital assets, user-generated content, and interaction histories must be persistently accessible yet resistant to censorship and tampering. Traditional cloud storage solutions, while efficient, centralize control and expose user data to potential breaches or unilateral service disruptions. Decentralized storage networks address these challenges by distributing data across a network of peers, leveraging cryptographic techniques to ensure data integrity and availability.

InterPlanetary File System (IPFS) is one of the most widely adopted decentralized storage protocols. It operates by identifying files through content-addressed hashes, enabling immutable and verifiable references to data without reliance on specific network locations. Filecoin, built atop IPFS, introduces an incentive layer that rewards nodes for storing data, thereby supporting long-term persistence through market-based mechanisms. Other platforms, such as Arweave and Storj, offer alternative models, including permanent archiving and encrypted file sharing.

In the Metaverse, decentralized storage is essential for hosting large-scale 3D assets, virtual environments, and media files that users interact with. Ensuring that such data is accessible regardless of platform or location contributes to the interoperability and continuity of user experiences. Moreover, the use of decentralized storage strengthens the guarantees of content authenticity and resistance to data manipulation, aligning with the broader goals of trust and transparency in Web 3.0 applications.

Nonetheless, decentralized storage raises significant regulatory and technical questions, particularly in relation to data protection frameworks such as the GDPR. The immutable nature of content addressing complicates the implementation of rights such as data erasure and correction, calling for hybrid solutions that combine off-chain data indexing with user-controlled access mechanisms.

### 3.5 Oracles and external data integration

While blockchain networks excel at providing secure and deterministic computation, they are inherently isolated from the external world. This limitation prevents smart contracts from directly accessing real-world information, such as asset prices, weather conditions, or sensor outputs, which is often essential for dynamic application behavior. Oracles serve as intermediaries that fetch, verify, and relay off-chain data to blockchain environments, thereby extending the functional reach of decentralized applications.

The integration of oracles enables a wide range of use cases. In financial applications, oracles supply accurate price feeds that underlie derivatives contracts, automated lending protocols, and stablecoins. In supply chain contexts, they can ingest data from IoT devices to confirm delivery times, monitor environmental conditions, or verify provenance claims. In the Metaverse, oracles can dynamically modify digital environments based on real-world events or link in-game assets to physical performance metrics.

However, the use of oracles introduces new trust and security considerations. Because smart contracts rely on oracles for input, any manipulation or failure at the oracle level can compromise the contract’s execution. This vulnerability, often referred to as the “oracle problem,” is addressed through techniques such as decentralized oracle networks, which aggregate data from multiple sources to improve reliability and resilience. Chainlink is one of the most prominent implementations of such a network, offering cryptographic proofs and economic incentives to ensure data accuracy and availability.

The inclusion of oracles in Web 3.0 architecture underscores the need for robust data governance and standardization practices, especially as data feeds become increasingly complex and integral to high-value transactions and automated decisions.

### 3.6 Interoperability protocols

Interoperability is a fundamental requirement for realizing the full potential of Web 3.0 and the Metaverse, where users, digital assets, and applications must be able to move seamlessly across heterogeneous platforms and networks. The current Web 3.0 landscape is characterized by fragmentation, with multiple blockchains operating in silos and employing incompatible standards. This lack of interoperability inhibits asset portability, complicates identity management, and limits the scalability of decentralized services.

Several technological initiatives aim to address this challenge. Platforms such as Polkadot and Cosmos introduce architecture frameworks that enable cross-chain communication and asset transfer through shared protocols and interoperability layers. These solutions rely on specialized components—parachains, hubs, bridges—that translate and validate transactions between otherwise incompatible networks. At a higher level, interoperability is also being pursued through standardization efforts. Ethereum’s ERC standards, for example, define token behavior in a consistent manner, facilitating interaction across a wide range of decentralized applications.

Within the Metaverse, interoperability extends beyond blockchain protocols to encompass 3D rendering formats, avatar systems, virtual economies, and identity frameworks. The Metaverse Standards Forum, involving both Web 3.0-native and traditional technology companies, is working toward defining open specifications for shared virtual environments. This includes coordination around file formats (such as glTF and USD), interaction protocols, and spatial computing interfaces.

Achieving meaningful interoperability also requires legal and institutional coordination. Issues related to digital rights management, cross-border data transfer, and jurisdictional authority must be resolved to support the seamless operation of decentralized systems at a global scale. As such, interoperability is not only a technical goal but also a socio-legal imperative that lies at the heart of a sustainable and inclusive digital future.

## 4 Use cases and value generation

Although Web 3.0 is still in its early stages, many companies are already experimenting with various ways to generate value using Web 3.0 tools. These initiatives represent not only technological advancements but also a new approach to business strategy. Some companies are beginning to adopt Web 3.0 tools to gain deeper insights into consumer behavior, enhancing customer journey traceability and participating in customer communities. This enables improved customer experience through more personalized products and services. These efforts also contribute to brand building and increase transparency in product sourcing, manufacturing, and distribution, thereby improving corporate governance and sustainability credentials.

The advent of Web 3.0 ushers in a new era of opportunities and challenges for companies operating in the information technology sector. In the near future, organizations will increasingly need to update and redesign their web presence. Web 3.0 is not merely an upgrade, it represents a fundamental shift towards greater personalization.

Typically, the emergence of new technologies creates two distinct pathways for existing enterprises: the sustainability path, which involves adopting new technologies while maintaining core business models and improving existing operations; the disruptive path, which leads to entirely new business models and often results in the delivery of innovative solutions to users.

Web 3.0 adoption follows a similar framework, presenting both challenges and opportunities. Value creation, the elimination of central authorities, and enhanced security and trust offer multiple ways to redefine business models. Web 3.0 embodies a transformative phase of internet development, rooted in the tenets of decentralization, autonomy, and security. Its effective deployment necessitates an integrated framework encompassing three fundamental dimensions: technological innovation, economic models, and legal structures. The technological pillar centers on cutting-edge advancements such as blockchain, artificial intelligence, and decentralized peer-to-peer systems, which are vital for constructing a robust and dependable Web 3.0 infrastructure. The economic pillar pertains to the digital economy and incentive mechanisms, requiring a deep understanding of how economic dynamics shape the development and adoption of Web 3.0, while ensuring that incentives are aligned with the ecosystem's objectives. Lastly, the legal pillar emphasizes the protection of personal data and the security of the data chain, with privacy computing and blockchain regulation serving as critical considerations. The successful maturation of Web 3.0 hinges on the synergistic progression of these dimensions, as any disparity among them could jeopardize the integrity of the entire ecosystem.

### 4.1 Legal foundations: Privacy and the data chain

The legal pillar of Web 3.0 focuses on privacy computing and the data chain. Given Web 3.0's foundation in decentralization and user autonomy, privacy is crucial to ensuring that users maintain control over their data and that it remains secure. Privacy computing provides multiple advantages, including the protection of personal data from third-party misuse, enhanced user control over data sharing and usage, transparency in data collection and analysis, and robust security against unauthorized access and cyberattacks.

There are currently three main categories of privacy computing technologies:

1. Secure Multi-party Computing (SMC): Allows multiple parties to jointly analyze data without revealing their private inputs.
2. Federated Learning (FL): Enables training of machine learning models on distributed datasets without centralizing data.
3. Trusted Execution Environment (TEE): Provides secure enclaves for executing code and storing sensitive data.

Privacy computing supports user data protection across the data chain:

- First Mile: Focuses on data provenance and identifying the responsible entity (e.g., sensor, institution) ( Simmhan *et al.*, 2005). Legal frameworks must define rights and responsibilities, enforce data-use agreements (possibly via smart contracts), and use blockchain for provenance and digital signatures for authenticity.
- Middle Mile: Ensures secure data transfer. Legal frameworks should govern access rights and compliance with privacy regulations. Smart contracts can automate access control, though blockchain’s immutability complicates rights like the right to be forgotten under GDPR ( Moreno, 2019). Encryption is essential here.
- Last Mile: Defines how final data is used and protected. The end user must retain control and be informed. Measures such as data pseudonymization and anonymization, transparent privacy policies, and user-informed consent are critical.

**Figure 1.**
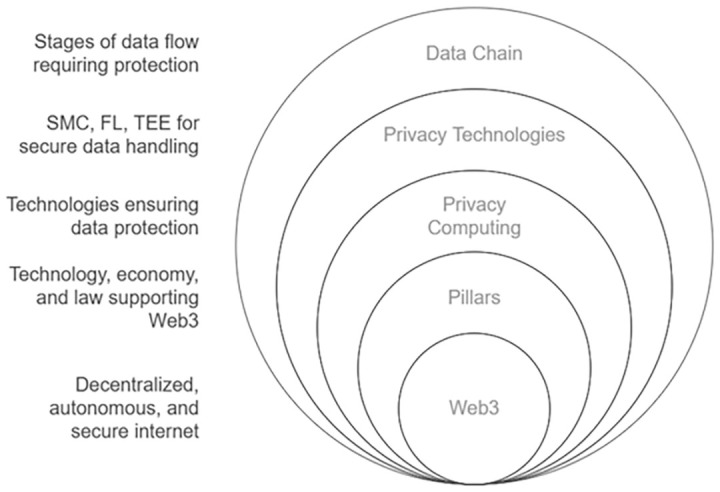
Web 3.0 Ecosystem and Privacy.

Thus, a comprehensive legal framework should be established to address several critical aspects (see Figure 1). It must guarantee data provenance and accountability to ensure the traceability and responsibility of data origins, which builds trust among users and stakeholders by verifying the authenticity of transactions and data sources on decentralized networks, thereby enhancing the reliability of blockchain systems. It should also define the rights and responsibilities of all data stakeholders to create clear expectations and obligations, fostering a fair and transparent ecosystem where participants are incentivized to engage responsibly, driving adoption and collaboration in Web3 environments.

Additionally, the framework needs to ensure data integrity and security during transit to protect against tampering or breaches, which is essential for maintaining the immutability and trustworthiness of blockchain records, thus increasing user confidence and the perceived value of decentralized solutions. It must enforce the right to be forgotten, allowing individuals to have their data erased under certain conditions, which aligns with user empowerment principles of Web3, adding value by prioritizing personal autonomy and privacy in digital interactions. Furthermore, access should be restricted to authorized parties only to prevent unauthorized use, safeguarding sensitive information and reinforcing the secure nature of blockchain technology, which in turn attracts businesses and users seeking robust data protection. The framework should also specify the final usage of data and the protections in place to govern its application, ensuring that data handling aligns with ethical standards and intended purposes, thereby creating a predictable and value-driven environment for innovation in Web3 applications. Lastly, it must safeguard end-user privacy to uphold trust and confidentiality within the system, a cornerstone of Web3’s value proposition, as it differentiates decentralized platforms from traditional centralized systems by prioritizing user control and security, ultimately driving engagement and long-term sustainability of blockchain ecosystems.

## 5 Managerial insights and future developments

Traditional models of innovation diffusion and management are inadequate for fully capturing the nuanced dynamics of the digital economy, where Web 3.0 and the Metaverse play pivotal roles as transformative paradigms. While academic literature on these topics remains relatively sparse, several critical themes and insights are beginning to crystallize, shedding light on the unique challenges and opportunities presented by these emerging domains.

First, both Web 3.0 and the Metaverse are fundamentally reliant on decentralized infrastructure as a core architectural principle. Blockchain, along with the broader category of Distributed Ledger Technologies (DLTs), serves as the most suitable foundational backbone due to its inherent properties of transparency, immutability, and trustlessness. However, achieving effective deployment and scalability necessitates seamless integration with a suite of complementary technologies, including the Internet of Things (IoT) for real-world data connectivity, Digital Twins for virtual replication of physical assets, Artificial Intelligence (AI) for intelligent decision-making and automation, Edge Computing for low-latency processing, and advanced telecommunications networks such as 5G and emerging 6G for high-speed, reliable connectivity. This multifaceted integration, while technically complex and demanding in terms of interoperability and standardization, unlocks significant opportunities for Information and Communication Technology (ICT) and telecommunications firms. These opportunities are particularly pronounced in vertical sectors such as port logistics, dry ports (as evidenced by initiatives like 5G-LOGINNOV, 2024), and last-mile delivery, where decentralized systems can optimize supply chain transparency, enhance operational efficiency, and reduce costs through real-time data sharing and automation.

Second, the advent of Web 3.0 and the Metaverse necessitates the redefinition of economic interactions between users, consumers, and stakeholders within digital ecosystems. Traditional diffusion models, such as the Bass model, are predicated on assumptions rooted in Chronos—linear, predictable timeframes and repeatable patterns of consumption characteristic of conventional economies. By contrast, Web 3.0 and the Metaverse derive their value from Kairos, a concept emphasizing singular, qualitative moments of user interaction and unique, immersive experiences that defy linear progression. This fundamental shift in value creation—from replicable transactions to personalized, irreplaceable engagement—demands the development of novel network-based economic models, interoperable standards, and supporting technologies. These new frameworks must account for tokenized incentives, decentralized governance via Decentralized Autonomous Organizations (DAOs), and user-driven content creation, which collectively redefine how value is generated, distributed, and sustained in virtual environments.

Currently, a formalized use case framework for Web 3.0 and Metaverse applications remains absent, with most initiatives still in experimental phases, pending broader market validation and adoption. Many of these pilots are exploratory, testing the boundaries of user engagement, technological feasibility, and economic viability in nascent ecosystems. This gap highlights an urgent need, akin to prior observations in non-crypto blockchain contexts, for structured feasibility analyses and robust deployment methodologies tailored specifically to Web 3.0 and Metaverse projects (e.g., Perboli *et al.*, 2018). Such methodologies should encompass technical readiness assessments, economic impact evaluations, and legal compliance checks to mitigate risks associated with scalability, interoperability, and regulatory uncertainty. Furthermore, they should incorporate stakeholder mapping to identify key actors—ranging from developers to end-users—and align their incentives to ensure sustainable ecosystem growth. Without these structured approaches, the potential of Web 3.0 and the Metaverse to redefine digital interaction risks being curtailed by fragmented efforts and unaddressed foundational challenges.

### 5.1 Data economy and system sustainability

While there is a broad consensus among analysts that data marketplaces will generate substantial economic value within the digital economy, a coherent and universally accepted paradigm for data valuation—encompassing both economic and ethical dimensions—remains elusive. Web 3.0, with its inherent characteristics of openness, decentralization, and participatory governance, holds the potential to serve as a powerful catalyst in addressing this gap. By leveraging blockchain and distributed ledger technologies, Web 3.0 can facilitate transparent data ownership, enable peer-to-peer data trading through tokenized incentives, and empower users to control the monetization of their personal information. However, realizing this potential requires overcoming significant conceptual and practical hurdles to ensure that data marketplaces are both equitable and sustainable.

A critical prerequisite is the development of a sustainable and economically viable model for data marketplaces, one that meticulously balances multiple dimensions of sustainability: energy consumption, social impact, and operational feasibility. Energy sustainability is particularly pressing given the historically high-power demands of blockchain networks, necessitating the adoption of energy-efficient consensus mechanisms like Proof of Stake (PoS) over energy-intensive Proof of Work (PoW). Social sustainability involves ensuring fair access and benefits for all participants, preventing monopolistic control by large entities, and safeguarding user privacy. Operationally, the model must support scalable infrastructure capable of handling vast data transactions without compromising performance or security. In this context, collaborative efforts are underway, notably through initiatives like the NOUS project under Horizon Europe, where TIM and the POLITO group are partnering with the European Commission ( NOUS consortium, 2024). Their work focuses on defining a European cloud infrastructure and establishing standardized frameworks that can underpin secure, interoperable, and sustainable data marketplaces across the continent, ensuring alignment with European values and regulations such as GDPR.

Despite the promise, several barriers impede the realization of data marketplaces as a cornerstone of Web 3.0. One prominent challenge is investor short-termism, which poses a significant hurdle to the necessary long-term commitment. Data marketplaces demand substantial upfront investment in infrastructure, technology development, and regulatory compliance, yet the return on investment (ROI) often materializes only over a medium to long-term horizon. This mismatch discourages risk-averse or short-sighted investors who prioritize quick returns over strategic, foundational advancements, potentially stalling the growth of innovative data ecosystems.

Another pressing concern is data authenticity, especially as data marketplaces rely on the integrity of information sourced from diverse inputs. Pilot projects, such as those conducted under the CONCORDIA consortium focusing on cybersecurity, have illuminated the vulnerabilities of IoT sensor networks, which are increasingly integral to data collection in Web 3.0 environments ( CONCORDIA, 2024). These studies reveal that IoT devices are susceptible to cyberattacks that can inject malicious or falsified data into blockchain systems, undermining their immutability and trustworthiness—a core value proposition of blockchain technology. As a mitigation strategy, Cellular IoT is being explored for its robust security features, including the ability to certify critical metadata such as timestamps, geolocation, and software signatures of data sources. This certification enhances traceability and authenticity, reducing the risk of data manipulation and bolstering confidence in decentralized data marketplaces.

From a technical perspective, the integration of advanced computational methodologies is crucial for optimizing Web 3.0 and blockchain-integrated services. AI-based surrogates for complex optimization models are emerging as a viable solution, offering significantly faster response times compared to traditional optimization techniques, albeit at the cost of slightly lower accuracy. These AI-driven approaches are particularly well-suited for real-time, demand-driven systems such as e-commerce platforms or dynamic blockchain services, where speed is often prioritized over precision. In contrast, classic optimization methods, while more accurate, are frequently computationally intensive and incompatible with the resource constraints of on-chain smart contract execution, which requires lightweight and efficient algorithms to operate within the decentralized environment's limitations ( Bruni *et al.*, 2023).

Additionally, wallet-based applications—key interfaces for user interaction with Web 3.0 and blockchain ecosystems—face significant usability challenges that hinder mainstream adoption. The development tools for creating these applications remain fragmented, with inconsistent standards and incompatible frameworks complicating the process for developers. For end-users, the experience is often marred by technical complexity, including the need to manage private keys, understand gas fees, and navigate unintuitive interfaces. This steep learning curve alienates non-technical users, limiting the accessibility and appeal of decentralized applications (dApps). Addressing these barriers requires concerted efforts to standardize development tools, streamline user interfaces, and enhance educational resources to demystify blockchain technology, thereby fostering broader engagement with Web 3.0 ecosystems.

Thus, while data marketplaces hold immense potential to drive economic value in the Web 3.0 era, their success depends on addressing multifaceted challenges spanning sustainability, investment models, data integrity, technical optimization, and user experience. Collaborative initiatives, innovative technologies like Cellular IoT and AI, and a long-term vision are essential to creating a robust, ethical, and accessible data economy within the decentralized landscape of Web 3.0 and the Metaverse ( Perboli *et al.*, 2020; Perboli *et al.*, 2025).

### 5.2 NFTs and the issue of trust

There is increasing interest in leveraging NFTs to tokenize both digital and physical goods, reflecting a broader trend toward asset digitization in Web 3.0 ecosystems. However, several critical concerns and unresolved challenges rather than providing definitive solutions, underscoring the need for cautious and informed approaches to NFT implementation.

First, the integrity of blockchain systems, which underpins the functionality of NFTs, fundamentally relies on the assumption of honest majority behavior among network participants. The most vulnerable point in this process is the "first mile", the initial stage of data generation, where inaccuracies or manipulations can compromise the entire chain of trust. For instance, data input at the source could be falsified before being recorded on the blockchain, rendering subsequent immutability moot. To mitigate this risk, emerging technologies such as Cellular IoT offer promising solutions by certifying critical metadata like location and timestamp, while also incorporating cryptographic guarantees to ensure data authenticity ( Capocasale *et al.*, 2021). These mechanisms enhance the reliability of the data origination process, strengthening the foundation upon which NFTs and other blockchain-based assets are built, though they do not fully eliminate the risk of malicious intent at the source.

Second, NFTs introduce significant trust challenges, particularly concerning the behavior of centralized entities involved in the process, such as artists, certifiers, or issuing authorities. These entities could engage in deceptive practices that undermine the perceived uniqueness and value of NFTs. For example, they might mint duplicate NFTs across different blockchain networks, creating multiple claims to the same asset and diluting its scarcity—a core driver of NFT value. They could also make imperceptible alterations, such as a one-pixel change in a digital artwork, to create technically distinct but visually identical tokens, deceiving buyers about originality. Additionally, sequential issuance and delayed revelation of NFTs could erode trust, particularly in high-stakes applications like patent certification, where timing and transparency are critical. These issues raise a fundamental question: if trust in issuers is a prerequisite for NFT legitimacy, does the use of blockchain add meaningful value over traditional systems? For instance, if we trust a university to issue diplomas as NFTs, why not trust it to maintain a centralized database instead? The distinction lies in blockchain's immutability, which prevents retroactive manipulation of records—an advantage over centralized systems prone to tampering or data loss. However, whether this benefit justifies the complexity and cost of NFTs varies by use case, requiring careful evaluation of trade-offs between decentralization and practicality.

Third, associating NFTs with physical goods presents substantial challenges in aligning digital and physical ownership. Unlike purely digital assets, where ownership is solely governed by blockchain records, physical goods exist in a tangible realm subject to real-world constraints like theft, loss, or legal disputes over possession. Establishing a reliable link between an NFT and a physical item, such as artwork, real estate, or luxury goods, often requires intermediaries or additional legal frameworks to enforce ownership claims, which can reintroduce centralization and undermine the decentralized ethos of Web 3.0. Moreover, discrepancies between digital records and physical reality, such as when an NFT is transferred but the corresponding physical item is not, can lead to conflicts that blockchain alone cannot resolve. This disconnect highlights a significant limitation in the current application of NFTs to physical assets, suggesting that robust off-chain mechanisms, such as escrow services or IoT-enabled tracking, are necessary to bridge this gap and ensure trust in tokenized physical goods.

Beyond these specific concerns, NFTs face broader technical limitations that temper their utility and scalability. While they have proven valuable in niche applications, such as digital art, collectibles, and virtual real estate within the Metaverse, their widespread adoption is often driven more by market hype and speculative fervour than by technical robustness or intrinsic utility. The revenue generated from NFT transactions frequently hinges on perceived value, fueled by cultural trends and social media buzz, rather than on the underlying strength or security of technology. This perception-driven market dynamic leaves NFT ecosystems vulnerable to volatility and diminishes long-term sustainability. Moreover, the majority of users engaging with NFTs remain unaware of the inherent trade-offs and complexities involved, such as high transaction fees (gas costs on networks like Ethereum), environmental concerns related to energy-intensive blockchains, and the risk of loss due to wallet mismanagement or phishing attacks. This knowledge gap among participants exacerbates systemic risks and raises ethical questions about the responsibility of developers and platforms to educate users on the limitations and potential pitfalls of NFT engagement. While NFTs offer innovative possibilities for tokenizing digital and physical goods, their implementation is fraught with critical challenges related to blockchain integrity, issuer trust, alignment with physical assets, and technical constraints. The hype surrounding NFTs often overshadows these underlying issues, with market dynamics prioritizing perceived value over practical utility. For NFTs to realize their transformative potential within Web 3.0, stakeholders must address these concerns through enhanced technological solutions, transparent practices, and user education, ensuring that adoption is grounded in substantiated value rather than transient enthusiasm.

### 5.3 Towards a unified legal-economic-technological framework

As AI, Web 3.0, and the Metaverse become increasingly intertwined within economic, political, and social systems, the urgent need to govern their global development and convergence grows more pressing. Unlike traditional technologies with primarily local or national impacts, these digital paradigms transcend borders, generating complex externalities that no single country can address unilaterally. The regulation of decentralized Web 3.0 infrastructures and immersive Metaverse environments, international cooperation is indispensable to align these technologies with democratic values, sustainable development goals, and equitable access. This section presents a unified governance framework integrating the key dimensions of Web 3.0 and the Metaverse to highlight their overlapping challenges and the need for a unified, adaptive regulatory framework.

***5.3.1 A cross-border vision for AI, Web 3.0, and the metaverse***

While regional regulations, such as the EU AI Act, Data Act, and GDPR, provide critical foundations for managing digital technologies, they fall short in addressing transboundary issues inherent to Web 3.0, and the Metaverse, including decentralized data flows, cybersecurity threats, algorithmic bias, and the integrity of blockchain-based systems ( European Parliament, 2016; European Parliament, 2023; European Parliament, 2024). The current landscape of fragmented governance across these domains’ risks amplifies global inequalities, enabling regulatory arbitrage where entities exploit jurisdictional differences, and undermining efforts to establish cohesive ethical standards and accountability mechanisms. For instance, Web 3.0’s reliance on decentralized networks like blockchain complexifies traditional regulatory oversight, while the Metaverse introduces novel challenges in virtual identity protection, property rights, and cross-platform interoperability. Against this backdrop, a new phase of governance is required—one that fosters coherence across jurisdictions, embraces diversity in normative traditions, and integrates the unique attributes of Web 3.0, and the Metaverse into a holistic framework.

Major global players such as China, the EU, and the US are central to shaping this multifaceted governance debate, with their distinct regulatory models, precautionary, market-driven, and state-centric, reflecting diverse legal cultures and political economies. Despite these differences, there is growing recognition among policymakers, academics, and industry stakeholders of the need for a shared governance framework that spans AI, decentralized technologies, and virtual environments ( Perboli *et al.*, 2025).

The EU actively promotes its regulatory model as a global benchmark, with initiatives like the AI Act, the Data Act, and the GDPR, influencing international discourse through its risk-based approach and focus on fundamental rights ( Heymann *et al.*, 2023). The EU’s regulatory diplomacy, evident in engagements with the OECD, G7, and United Nations, extends to Web 3.0 and Metaverse contexts through policies like the Digital Markets Act and GDPR, aiming to create a rule-based digital environment grounded in European values. The EU’s regulatory framework covers virtual currencies, initial coin offerings (ICOs), and the legal validity of smart contracts ( Blemus, 2018). However, the applicability of this stringent, ex-ante regulation outside European contexts remains complex, with critics arguing it may stifle innovation in rapidly evolving fields like blockchain applications and Metaverse platform development.

The US maintains global leadership through technological prowess and influence over standard-setting bodies. Its soft power in digital governance stems from an open research ecosystem, academic leadership, and public-private collaborations, which also drive innovations in blockchain and Metaverse technologies. The US has adopted a fragmented regulatory approach, with both federal and state-level initiatives. The regulatory landscape is characterized by a mix of cautious support for innovation and concerns about consumer protection and financial stability ( Suda *et al.*, 2017). Its multi-stakeholder model, while promoting innovation and agility, contributes to normative fragmentation, particularly in Web 3.0, where inconsistent state-level policies on cryptocurrency and NFTs create regulatory uncertainty.

China has adopted a restrictive stance on blockchain-related financial activities, prioritizing control over decentralized systems. The Chinese government has imposed a complete ban on cryptocurrency trading and mining, driven by concerns over financial stability and consumer protection. Despite this, China is actively pursuing blockchain-based financial innovation under state supervision, with a significant focus on the development of a Central Bank Digital Currency (CBDC) known as the digital yuan. This initiative, led by the People’s Bank of China, aims to enhance financial inclusion and streamline the efficiency of cross-border payments. By prohibiting private cryptocurrency exchanges and mining operations, China has effectively dismantled the unregulated cryptocurrency market within its borders, channeling blockchain technology development toward state-controlled applications that align with national economic and regulatory objectives ( Hu, 2023).

China, meanwhile, aligns its global Web 3.0 strategies with domestic objectives, extending influence through the Belt and Road Initiative to export technologies and regulatory logic to partner countries. Its participation in international bodies like the International Telecommunication Union and AI for Good Summit promotes a vision of governance rooted in state sovereignty and developmental priorities, often prioritizing control over decentralized Web 3.0 ecosystems and Metaverse interactions ( Hu, 2023).

***5.3.2 Challenges in a global governance of Web 3.0 and the metaverse***

Integrating Web 3.0 and the Metaverse into global governance introduces additional layers of complexity beyond those of AI alone. Key challenges include:

1. **Decentralization vs. Regulatory Control**: Web 3.0’s decentralized architecture, powered by blockchain and distributed ledger technologies, inherently resists traditional centralized regulation, creating tensions with national legal frameworks that assume hierarchical authority. For instance, enforcing data privacy laws on a blockchain where data is immutable conflicts with principles like the right to be forgotten enshrined in GDPR.
2. **Virtual Jurisdiction in the Metaverse**: The Metaverse operates as a borderless, immersive space, raising questions of jurisdiction over virtual crimes, property disputes, and identity theft. Determining which laws apply to interactions in virtual worlds, especially when users and platforms span multiple countries, remains unresolved.
3. **Interoperability and Standardization**: Both Web 3.0 and the Metaverse require interoperable standards to ensure seamless user experiences across platforms and blockchains. Without global coordination, fragmented technical standards risk creating silos, undermining the decentralized ethos and user accessibility.
4. **Data Integrity and Trust in Tokenized Systems**: NFTs and other tokenized assets in Web 3.0 and the Metaverse face issues of data authenticity at the “first mile” of data generation, vulnerability to cyberattacks, and trust in issuers, necessitating novel security mechanisms like Cellular IoT for certification ( Capocasale *et al.*, 2021).

***5.3.3 Institutional mechanisms and stakeholder roles***

The governance of AI, Web 3.0, and the Metaverse at a supranational level requires institutional mechanisms that transcend state-centric models and embrace multi-level, polycentric architectures. Beyond existing bodies like the United Nations, OECD, and UNESCO, which lack formal enforcement powers, a proposed International Digital Technology Agency, as the International Atomic Energy Agency (IAEA), might oversee compliance, facilitate cooperation, and promote beneficial uses of these technologies. However, a purely top-down approach risks failure; it must be complemented by bottom-up institutional development, capacity-building in emerging economies, and continuous global dialogue to ensure fairness and legitimacy. For Web 3.0 and the Metaverse, decentralized governance models inspired by DAOs (Decentralized Autonomous Organizations) could also play a role, allowing community-driven rule-making while aligning with global principles through interoperable standards.

Stakeholder engagement remains critical, with specific roles for civil society, academia, and infrastructure providers in shaping integrated governance:

- **Civil Society**: Beyond governments and corporations, civil society organizations are vital for promoting democratic accountability in Web 3.0 and Metaverse governance, ensuring that virtual spaces reflect diverse perspectives, especially from marginalized groups. Mechanisms like participatory DAOs or virtual citizen assemblies could institutionalize their involvement.
- **Academia**: Academic institutions can conduct independent audits of AI algorithms, blockchain smart contracts, and Metaverse platforms, assessing impacts on privacy, equity, and security, while educating future developers and regulators on sustainable practices.
- **Compute and Blockchain Infrastructure Providers**: These entities act as intermediaries in the regulatory ecosystem, securing decentralized systems, enhancing visibility for policymakers through on-chain analytics, verifying user activities, and enforcing rules against violations. Their role is particularly crucial in ensuring compliance with data and privacy standards across Web 3.0 and Metaverse applications.

***5.3.4 Toward just, inclusive, and resilient governance across digital paradigms***

Global governance for Web 3.0, and the Metaverse demands more than abstract principles; it requires a coordinated, institutionally supported, and politically feasible architecture that addresses their convergence. As these technologies increasingly shape democratic processes, economic systems, and personal freedoms in both physical and virtual realms, there is a moral imperative to design frameworks that reflect pluralistic values, protect vulnerable communities, and prevent technological divides between the global North and South. The concentration of expertise, infrastructure, and investment in a few dominant countries raises concerns about dependency and inequality, particularly in the Metaverse, where access to high-end hardware and bandwidth limits participation for much of the Global South. Governance must incorporate redistributive mechanisms, such as technology transfer, capacity-building programs for blockchain literacy, and equitable access to virtual resources, with institutions like the World Bank and ITU playing key roles in ensuring shared benefits.

Regulations must account for where these technologies are developed and deployed, recognizing the unequal conditions under which they affect diverse societies. A multi-layered approach blending national sovereignty with international cooperation is essential, incorporating binding treaties on algorithmic and blockchain accountability, shared enforcement protocols for virtual environments, and global funding to support regulatory capacity in low-income regions. Moreover, the environmental impact of these technologies, especially energy-intensive blockchain networks and large-scale AI training for Metaverse applications, must be addressed through international standards for sustainability, such as carbon-aware design and mandated impact assessments ( Perboli *et al.*, 2025).

***5.3.5 Shaping unified vision***

Global governance for AI, Web 3.0, and the Metaverse stands at a critical juncture. The next decade will determine whether these technologies foster inclusive, sustainable development or exacerbate inequalities and erode democratic norms in both digital and physical spaces. The path forward requires bold institutional imagination, political will, and a shared commitment to fairness, safety, and human dignity across all domains. A unified governance framework, anchored in ethical principles and operationalized through inclusive institutions, offers the best chance to navigate the risks and maximize the rewards of this digital revolution. The question is not merely how to govern these technologies, but what kind of interconnected world, real and virtual, we aim to build through them.

A comprehensive legal framework must integrate with existing regulations. A key conflict arises between the right to be forgotten and blockchain’s technical immutability. One solution is a legal framework that prohibits use of data flagged for erasure, rather than deleting it technically. This raises further issues, such as using aggregated data (from users who requested deletion) in AI models powering essential services. These complexities demand a co-designed, multi-disciplinary, and multi-domain framework that is transparent, minimally invasive, and guided by Lean principles. The Lean Business approach, proven effective in innovation and blockchain systems, may serve as a useful model here ( Fadda *et al.*, 2018; Fadda *et al.*, 2022; Narayan *et al.*, 2024; Rauchs *et al.*, 2018).

## 6 Conclusions

Web 3.0 and the Metaverse represent a profound shift in how digital value is created, distributed, and governed. While still in their formative stages, these paradigms are reshaping the digital economy by introducing new principles of decentralization, user empowerment, and trustless infrastructure. The integration of blockchain, smart contracts, and self-custodial wallets lays the foundation for a more secure, transparent, and participatory web.

However, the successful development and adoption of Web 3.0 technologies require more than just technological innovation. As discussed, a holistic framework encompassing legal, economic, and technological dimensions is essential. The legal pillar, in particular, plays a central role in ensuring user rights, data privacy, and compliance with broader regulatory frameworks like the GDPR, AI Act, and Data Act. Without such alignment, the full potential of Web 3.0 may remain unrealized or become a source of legal and ethical controversy.

At the same time, businesses are confronted with both opportunities and risks. Web 3.0 offers novel ways to engage customers, personalize services, and build trust, but it also demands new governance models, robust privacy protections, and a reconsideration of traditional business strategies. The experimental nature of current use cases suggests the need for standardized methodologies for feasibility assessment, implementation, and value measurement.

As the digital economy evolves toward user-centric and data-driven models, issues such as data authenticity, sustainability, interoperability, and scalability become ever more critical. Emerging technologies such as AI, IoT, and Federated Learning must be orchestrated alongside Web 3.0 to address these challenges effectively.

Finally, the hype surrounding technologies like NFTs and the Metaverse must be met with critical scrutiny. Without a clear, transparent, and user-oriented governance structure, there is a risk of repeating the centralization and exploitation patterns that Web 3.0 seeks to overcome.

The future of Web 3.0 and the Metaverse will depend not only on technological progress but also on the ability of institutions, businesses, and users to co-create a trustworthy, inclusive, and sustainable digital ecosystem. Only through multidisciplinary collaboration, legal foresight, and technological responsibility can we ensure that the next iteration of the internet serves the public good while fostering innovation.

## Ethics and consent

Ethical approval and consent were not required.

## Disclaimer

The views expressed in this article are those of the author(s). Publication in Open Research Europe does not imply endorsement of the European Commission.

## Data Availability

No data were associated with this article.
