# Supplementary material for: Decentralizing the future: Value creation in Web 3.0 and the Metaverse
Source: Open Res Eur. 2026 Jan 14;5:226. Originally published 2025 Aug 6. [Version 2] doi: 10.12688/openreseurope.20906.2 (PMC12828255; doi:10.12688/openreseurope.20906.2)
Supplement: Peer review history

# Reviewer report: Reviewer response for version 2

**DOI:** 10.21956/openreseurope.24571.r68410

**Published:** 2026-01-28

**Authors:** Dimitrios G. Kogias

**Affiliations:** 1 University of West Attica, Athens, Greece

The manuscript offers a clear and readable overview of Web 3.0 and the Metaverse, with a strong focus on blockchain technology value creation, and regulatory and governance aspects, particularly in the European context. The paper is well structured, accessible to an interdisciplinary audience, and shows good familiarity with the relevant literature. The presented statements are generally accurate and appropriately supported by references. As a narrative synthesis, the article can serve as a useful entry point for readers interested in the technological, economic, and legal dimensions of emerging Web3 ecosystems.

 On the other hand, the positioning and scope of the paper would benefit from clarification. While the manuscript is presented as a review, it does not follow a systematic review approach or clearly define how the literature was selected. Additionally, the proposed technological–economic–legal framework is already mainly integrative rather than conceptually new. The authors should therefore either explicitly frame the paper as a *narrative or position paper*, or strengthen the review aspect by *clarifying scope and methodology*. In addition, the discussion of the Metaverse is largely *limited to blockchain and Web3-related technologies*. Since the Metaverse is a broader ecosystem that also relies on VR/AR/XR, real-time 3D engines and human–computer interaction, the authors should either broaden the technological perspective or clearly state that the focus is specifically on the Web3/blockchain dimension of the Metaverse and explain why this only matters to them.

 Addressing these points would improve the overall coherence and alignment between the paper’s claims and its actual scope.

Is the review written in accessible language?

Yes

Are all factual statements correct and adequately supported by citations?

Yes

Are the conclusions drawn appropriate in the context of the current research literature?

Partly

Is the topic of the review discussed comprehensively in the context of the current literature?

Partly

Reviewer Expertise:

My research focuses on distributed systems and blockchain, including permissioned and public blockchain infrastructures, smart contracts, and decentralized identity. I also work on data governance and regulatory aspects of blockchain systems, particularly in the context of privacy, digital identity, and EU digital regulation, with experience in applied and interdisciplinary Web3 research.

I confirm that I have read this submission and believe that I have an appropriate level of expertise to confirm that it is of an acceptable scientific standard, however I have significant reservations, as outlined above.

# Reviewer report: Reviewer response for version 2

**DOI:** 10.21956/openreseurope.24571.r68074

**Published:** 2026-01-22

**Authors:** Pasika Ranaweera

**Affiliations:** 1 University College Dublin, Dublin, Ireland

Authors have addressed the issues I highlighted. Status: Approved

Is the review written in accessible language?

Yes

Are all factual statements correct and adequately supported by citations?

Partly

Are the conclusions drawn appropriate in the context of the current research literature?

Partly

Is the topic of the review discussed comprehensively in the context of the current literature?

Partly

Reviewer Expertise:

NA

I confirm that I have read this submission and believe that I have an appropriate level of expertise to confirm that it is of an acceptable scientific standard.

# Reviewer report: Reviewer response for version 1

**DOI:** 10.21956/openreseurope.22617.r66714

**Published:** 2026-01-07

**Authors:** Fabio zambardino, Andrea Borroni

**Affiliations:** 1 New Vision University (Ringgold ID: 484451), Tbilisi, Tbilisi, Georgia; 2 Universita degli Studi della Campania Luigi Vanvitelli (Ringgold ID: 18994), Caserta, Campania, Italy

This contribution offers a timely, comprehensive, and insightful exploration of Web 3.0 and the Metaverse, skilfully weaving together technological, economic, and legal dimensions. The authors succeed in presenting a holistic perspective on the transformative potential of these paradigms, emphasizing the interplay between decentralization, user empowerment, data governance, and sustainability. The discussion is rigorous yet accessible, providing both a thorough technical analysis of blockchain, smart contracts, digital wallets, oracles, and interoperability, as well as a clear examination of the regulatory landscape, from GDPR to the AI Act and global governance considerations.

 The paper stands out for its balance between visionary outlook and grounded analysis. It acknowledges both the immense opportunities offered by Web 3.0 and the Metaverse and the practical challenges related to privacy, data authenticity, scalability, energy efficiency, and the nascent adoption of NFTs. Managerial and policy insights are seamlessly integrated, highlighting the importance of multidisciplinary collaboration and a co-designed approach to governance that ensures inclusivity, ethical standards, and long-term resilience.

 Structurally, the manuscript is elegant and logically organized, guiding the reader from foundational concepts through technological mechanisms, use cases, and managerial implications, and culminating in a thoughtful synthesis of conclusions and future directions. The narrative is coherent and persuasive, and the depth of analysis reflects a sophisticated understanding of both academic and practical dimensions of this rapidly evolving field.

 In conclusion, this paper represents a significant contribution to the literature on the topic. It is well-written, methodologically sound, and intellectually compelling.

 There are no suggestions for improvement and recommend acceptance without revisions.

Is the review written in accessible language?

Yes

Are all factual statements correct and adequately supported by citations?

Yes

Are the conclusions drawn appropriate in the context of the current research literature?

Yes

Is the topic of the review discussed comprehensively in the context of the current literature?

Yes

Reviewer Expertise:

Comparative Law and Technology

We confirm that we have read this submission and believe that we have an appropriate level of expertise to confirm that it is of an acceptable scientific standard.

# Response

**Published:** 2026-01-08

**Authors:** Guido PERBOLI

We thank the reviewer for their thorough analysis of our manuscript and the positive evaluation.

# Reviewer report: Reviewer response for version 1

**DOI:** 10.21956/openreseurope.22617.r61318

**Published:** 2025-10-14

**Authors:** Pasika Ranaweera

**Affiliations:** 1 University College Dublin, Dublin, Ireland

**Summary of the paper**

 The article is a narrative review that discusses Web 3.0 and the Metaverse and argues for a holistic, sustainability‑oriented framework spanning three pillars: technological, economic, and legal/governance. It surveys core building blocks: blockchain infrastructure, smart contracts, wallets and identity, decentralized storage, oracles, and links them to emerging business and use‑case avenues and emphasizes privacy‑preserving data handling and evolving regulation (e.g., GDPR, AI Act, Data Act). The paper positions Web 3.0 as decentralization and user‑sovereignty‑centric and treats the Metaverse as an immersive layer that integrates those capabilities.

**Strengths**

- Clear motivation and scope: the abstract and introduction consistently frame decentralization, data sovereignty, and sustainability as the through‑line of the review.
- Holistic organizing lens: the three‑pillar sustainability framing (technological, economic, legal/governance) is repeated throughout and is easy to reuse in practice.
- Plain‑language on‑ramp: the presence of a plain‑language summary makes the topic accessible to non‑specialists without sacrificing the academic discussion.
- Governance awareness: value creation is repeatedly tied to privacy, data‑chain integrity, and evolving regulation in the EU and beyond.
- Pragmatic risk signals: the article calls out first‑mile authenticity, NFT/token trust, and the danger of fragmented, siloed standards.

**Weaknesses**

- Lack of review methodology: as a review article, it does not provide even a brief methods section (databases searched, date window, inclusion/exclusion), which limits replicability.
- There are no notable figures or tables that present any comparisons or state-of-the-art analysis.
- Even though some challenges are presented, they are not discussed in depth, nor are any solutions envisaged to overcome them.
- Definition hygiene: “Web 3.0,” “Web3,” and “Metaverse” are at times blended or capitalized inconsistently, which risks conflation of concepts.
- Inconsistently referring to the paper as the chapter or paper.
- Measurement gap: the term “value creation” is advocated, but no concrete KPIs or evaluation checklist are provided across the three pillars (e.g., privacy/sovereignty indices, latency/UX, energy per transaction, token‑economy health, or compliance cost).
- Under‑developed networks/edge mapping: there is little concrete linkage to connectivity and edge deployment realities (identity/oracles/storage placement; slice/latency budgets).
- Limited operational guidance: while risks are noted (authenticity, standards), few actionable patterns are offered (sensor attestation, TEEs, DAO guardrails, interoperability profiles).

**Grammatical issues and typos (with suggested fixes)**

- Use “paper” rather than “chapter” when referring to the article itself. Example: replace “This chapter proposes …” with “This paper proposes …”.
- Avoid comma splices. Example: “Web 3.0 is not merely an upgrade, it represents …” → “Web 3.0 is not merely an upgrade; it represents …” or split into two sentences.
- Clarify referents and tighten phrasing. Example: “At the heart of Web 3.0 lies the concept of decentralization, individuals or single entities no longer own services …” → “At the heart of Web 3.0 lies decentralization: services are not owned by single entities but are provided by the network.”
- Standardize capitalization throughout for terms such as “Web 3.0,” “Web3,” and “Metaverse.”
- Prefer clear comparisons. Example: “an International Digital Technology Agency, as the IAEA …” → “an International Digital Technology Agency, akin to the IAEA …”

**Recommendations to improve the quality**

1. Add a short review‑methods box. In 5–7 lines, list databases searched, date range, search terms, inclusion/exclusion criteria, and the synthesis approach. This will make coverage more credible and citable.
2. Add at least 2 or 3 figures to explain the concepts, especially the framework of the three pillars.
3. Add tables to compare different aspects mentioned under Section 3, and summarize solutions to the presented challenges.
4. Include an interoperability table. Summarize by layer: identity (DIDs/VCs), assets (ERC‑721/1155/6551), data exchange (DIDComm/VC‑DM), metaverse formats (USD/glTF), and storage (IPFS/Filecoin/Arweave), and highlight gaps where standards are immature.
5. Lock definitions early and keep them consistent. Provide one paragraph that distinguishes “Web3” (blockchain‑native, tokenized) from “Web 3.0” (broader next‑web including semantic, AI, and immersive facets) and how each maps to Metaverse components; then standardize capitalization across the paper.
6. Provide an evaluation checklist (one page). For each pillar, list 3–5 KPIs with examples: Technological (end‑to‑end latency, attestation coverage, L2/rollup energy per transaction, storage durability), Economic (user acquisition/retention with token incentives, treasury runway, fee‑model sustainability), Legal/Governance (lawful bases for processing, data‑minimization score, cross‑border data‑flow posture, turnaround times for data‑subject rights).
7. Map architecture to deployment realities. Add a figure that places wallets/SSI, oracles, decentralized storage, and smart‑contract logic against edge/metro/cloud topologies and typical latency budgets for immersive use cases.
8. Deepen the “first‑mile trust” discussion. Expand authenticity into a concrete pattern: sensor attestation (TEE/TPM/secure elements), timestamping and location proofs, signed ingestion at the edge, audit trails, and packet‑forwarding integrity—plus how these bind to token minting/NFT provenance.
9. Add a regulatory‑mapping matrix. Map common components (wallets, NFTs/tokens, DAOs, data marketplaces, oracles) to GDPR, AI Act, Data Act, and related touchpoints (lawful basis, DPIA triggers, high‑risk AI categories, data‑localization constraints).
10. Tighten prose and structure. Implement the grammar fixes above and consider consistent sub‑headings for each pillar to improve scanability.

Is the review written in accessible language?

Yes

Are all factual statements correct and adequately supported by citations?

Partly

Are the conclusions drawn appropriate in the context of the current research literature?

Partly

Is the topic of the review discussed comprehensively in the context of the current literature?

Partly

Reviewer Expertise:

Metaverse, Blockchain, 6G, ....etc.

I confirm that I have read this submission and believe that I have an appropriate level of expertise to confirm that it is of an acceptable scientific standard, however I have significant reservations, as outlined above.

# Response

**Published:** 2026-01-08

**Authors:** Guido PERBOLI

Thank you for your thoughtful and constructive feedback. In response, we have improved the overall clarity of the paper by adding two figures and two tables to better illustrate the proposed framework. We have also corrected typographical errors and stylistic inconsistencies throughout the manuscript and updated the reference section accordingly.
